# Supplementary material for: Resource Consumption and Remuneration Aspects in Navigated Screw Fixation Procedures with or without Additional Sacroplasty for Fragility Fractures of the Sacrum—A Prospective Clinical Study
Source: J Clin Med. 2022 Oct 18;11(20):6136. doi: 10.3390/jcm11206136 (PMC9605172; doi:10.3390/jcm11206136)
Supplement: Supplementary file 1 [file jcm-11-06136-s001.zip › jcm-1888439-supplementary.pdf]

Title: Resource consumption and remuneration aspects in navigated screw fixation procedures with or without additional sacroplasty for fragility fractures of the sacrum – a prospective clinical study
Journal: Journal of Clinical Medicine
Authors: Horst Balling, MD[1, 2, 3], Boris Michael Holzapfel, MD, PhD[3], Wolfgang Böcker, MD[3], Joerg Arnholdt, MD[3].
Affiliation: [1]Department for Spine Surgery and Traumatology, Orthopaedische Fachklinik Schwarzach, Dekan-Graf-Str. 2-6, 94374 Schwarzach, Germany; [2]Center for Spine Surgery, Neckar-Odenwald-Kliniken gGmbH, Buchen, Dr.-Konrad-Adenauer-Str. 37, 74722 Buchen, Germany, [3]Department of Orthopaedics and Trauma Surgery, Musculoskeletal University Center Munich (MUM), University Hospital, LMU Munich, Marchioninistr. 15, 81377, Munich, Germany
E-mail: horst.balling@neckar-odenwald-kliniken.de; horstballing@yahoo.de

Online Resource 1 Data on baseline conditions and study results (patients' gender, age, fracture sites, fracture morphology, concomitant injuries of anterior pelvic ring, surgical duration, 3D-/2D-radiation dose, fluoroscopy time, cement amount applied, implant costs, and remuneration)

patient	group	age (years)	gender	sacral fracture sites (S1+S2)	transalar	transforaminal	central	anterior pelvic ring fracture	surgical duration (minutes)	3D-radiation dose (mGycm)	2D-radiation dose ($mGycm^2$)	fluoroscopy time (sec)	cement amount (cc)	implant costs (€/surgery)	reimbursement (€/surgery)
1	NSF	90	f	1+0		1		yes	68	2058.95	818.04	29.11		166.30	6006.96
2	NSF	71	f	2+0	2			no	76	1269.46	1070.15	25.78		169.20	3507.54
3	NSF+ASP	75	f	1+0	1			yes	81	1269.46	4288.81	84.42	1.5	514.69	7253.05
4	NSF+ASP	74	f	2+0	2			yes	118	1822.89	4295.81	56.84	1.2	663.89	6402.97
5	NSF+ASP	79	f	2+0	2			no	93	2058.95	24799.23	60.5	1.2	522.59	6691.39
6	NSF	77	f	1+0	1			no	78	900.50	9784.50	27.14		79.60	5352.50
7	NSF	85	f	2+0	2			yes	139	1080.60	8643.38	55.51		251.70	6204.72
8	NSF+ASP	65	f	2+0	2			yes	136	450.25	5017.14	41.87	3.5	653.60	5555.82
9	NSF+ASP	77	f	2+2	4			no	211	899.35	19780.97	53.67	6	759.32	5555.82
10	NSF	80	f	2+0	1	1		yes	120	720.40	6554.71	32.51		173.40	5138.67
11	NSF	73	m	1+0			1	no	107	720.40	7355.94	34.02		173.40	6448.83
12	NSF	86	f	2+0		2		no	85	720.40	2846.82	23.05		173.40	6473.55
13	NSF	66	f	2+0	2			yes	80	720.40	6446.62	25.45		169.20	5555.82
14	NSF	58	f	1+0	1			yes	47	530.28	3292.94	15.69		156.30	5642.76
15	NSF	36	f	2+0	1	1		yes	123	2058.03	25332.20	46.20		172.00	5642.76
16	NSF+ASP	66	m	2+0	2			yes	110	1125.63	6368.78	36.96	3	557.20	6742.62
17	NSF+ASP	69	f	2+0	2			no	112	840.47	6700.20	35.18	1.5	523.29	5642.76
18	NSF+ASP	71	f	2+0	2			yes	109	870.49	3627.76	26.61	1.5	519.69	10807.32
19	NSF+ASP	84	f	2+0	2			no	121	840.47	8733.32	44.12	3	574.70	8381.29
20	NSF+ASP	85	f	1+0	1			yes	113	839.55	6635.32	33.38	1.5	520.49	10807.32
21	NSF+ASP	83	f	2+0	2			yes	100	870.49	3458.19	33.45	3	558.60	10807.32
22	NSF	73	f	1+0	1			no	58	1714.64	7197.66	29.39		156.30	5642.76
23	NSF	72	f	2+0	2			yes	49	570.32	3118.13	22.68		173.40	7408.91
24	NSF+ASP	78	f	1+0	1			no	105	630.35	2845.33	36.88	1.5	520.49	10807.32
25	NSF	68	m	2+0	2			yes	101	900.50	7417.13	36.63		173.40	5642.76

Title: Resource consumption and remuneration aspects in navigated screw fixation procedures with or without additional sacroplasty for fragility fractures of the sacrum – a prospective clinical study
Journal: Journal of Clinical Medicine
Authors: Horst Balling, MD[1, 2, 3], Boris Michael Holzapfel, MD, PhD[3], Wolfgang Böcker, MD[3], Joerg Arnholdt, MD[3].
Affiliation: [1]Department for Spine Surgery and Traumatology, Orthopaedische Fachklinik Schwarzach, Dekan-Graf-Str. 2-6, 94374 Schwarzach, Germany; [2]Center for Spine Surgery, Neckar-Odenwald-Kliniken gGmbH, Buchen, Dr.-Konrad-Adenauer-Str. 37, 74722 Buchen, Germany, [3]Department of Orthopaedics and Trauma Surgery, Musculoskeletal University Center Munich (MUM), University Hospital, LMU Munich, Marchioninistr. 15, 81377, Munich, Germany
E-mail: horst.balling@neckar-odenwald-kliniken.de; horstballing@yahoo.de

26	NSF+ASP	55	f	2+2	4		no	135	300.17	6510.23	55.61	6	641.02	6569.60
27	NSF+ASP	86	f	2+2	4		no	133	262.46	6843.39	71.38	3	574.70	11778.40
28	NSF+ASP	76	f	2+0	2		yes	100	262.65	3792.72	37.46	3	574.70	11778.40
29	NSF	73	f	2+0		2	no	52	1372.64	5363.93	20.89		173.40	5775.26
30	NSF+ASP	76	f	2+0	2		no	111	243.89	4964.16	49.48	3	558.60	7676.47
31	NSF+ASP	74	f	2+2	4		no	115	262.65	2497.26	31.48	3	575.40	8913.60
32	NSF+ASP	77	f	1+0	1		no	98	243.89	3691.61	31.62	3	558.60	6569.60
33	NSF+ASP	86	f	0+2	2		yes	105	1822.89	13374.70	47.74	1.5	558.60	11778.40
34	NSF	89	f	1+0	1		yes	48	568.28	6458.80	26.44		158.10	5775.26
35	NSF	80	f	2+2	4		no	152	155.54	6798.43	54.85		255.10	6569.60
36	NSF	90	f	2+2	4		yes	91	167.97	2537.18	32.61		172.00	6891.81
37	NSF	71	f	2+2	4		no	136	800.70	9762.73	60.35		258.00	7367.33
38	NSF	81	m	2+0	2		no	105	225.13	5096.97	44.75		172.70	6803.86
39	NSF	86	f	1+0	1		no	97	119.96	6105.66	46.82		173.40	8942.43
40	NSF	66	f	2+2	4		no	128	118.71	3041.91	30.21		172.00	7008.19
41	NSF	78	f	2+2		4	no	122	187.60	5404.84	56.83		245.20	5020.99
42	NSF+ASP	86	f	2+2	4		no	176	126.07	4005.34	44.49	7	764.93	7008.19
43	NSF	64	f	2+2		4	no	167	142.48	5637.84	41.89		258.00	7008.19
44	NSF+ASP	69	f	1+1	2		no	132	94.64	8415.65	53.31	3	608.49	7008.19
45	NSF	70	f	2+2	2	2	no	131	2745.27	6066.52	15.67		421.48	4924.68
46	NSF	84	f	1+0	1		yes	63	225.13	1186.75	7.4		266.87	13144.47
47	NSF	82	f	2+2	4		yes	81	1098.11	1364.67	8.27		399.03	11296.24
48	NSF+ASP	84	f	2+2	4		yes	124	141.08	5353.59	33.57	3	864.88	13456.99
49	NSF+ASP	85	f	2+2	4		no	112	1372.64	18847.3	102.3	7	1306.38	6837.37
50	NSF+ASP	76	f	2+2	4		no	115	188.86	14310.70	48.3	3	1333.93	6837.37
51	NSF+ASP	89	f	1+1	2		yes	102	1079.68	3681.50	17.7	3	709.92	4609.61
52	NSF+ASP	87	f	1+1	2		yes	117	184.14	7517.22	43.9	3	867.02	12539.07

S1 indicates sacral vertebra I; S2, sacral vertebra II; mGycm, milligray-centimeter; 3D-/2D-, 3-dimensional-/2-dimensional-; sec, seconds; cc, cubic centimeter; NSF, navigation-assisted screw fixation; NSF+ASP, navigation-assisted screw fixation and additional sacroplasty
